# More Deaths from Chloroform

**Published:** 1850-04

**Authors:** 


*More Deaths from Chloroform.*—Communicated for the Boston Med. and Surg. Journal, by Prof. John C. Warren.

Within the last few months an additional number of deaths have occurred from the use of chloroform. The whole number known and published hos now reached at least eigheen; but it is fair to suppose that many have occurred under such circumstances that the veil of secresy could be drawn over them.

The first of those in the following arrangement is quite remarkable for the striking character of its facts, and not less so for the clear and satisfactory manner in which they are related. Many practitioners in England, some inFrance, and a greater number in this country, have discontinued the employment of chloroform. In the mean time ether is used freely, daily and harmlessly. No ill consequence worthy of record has fallen within our knowledge; but, on the contrary, its usefullness becomes more obvious, in proportion to our experience of it, and multitudes of patients, as well as surgeons, are constantly pouring out their thanks for the discovery of so valuable and unexpected a mitigation of human suffering.

The uniform success of ether in this part of the country may be attributed to two circumstances: one is, its very free and decided use. The other is, the great caution exercised before and during its administration, and its consequent rejection from trifling operations and improper cases.

Sulphuric and strong chloric ether are both employed. The latter is most agreeable, but when freely used is apt to produce soreness of face, unless some unctuous substance has been previously applied.

I. *Instance of sudden Death from the use of Chloroform.* Reported to the Academie de Medecine, &c. Gazette Medicale de Paris, 20th October, 1849.

Madame Labrune, 33 years of age, in good health, applied to M. de Confevron with the request that he would administer chloroform to her previously to the extraction of a tooth, which the dentist said would be effected with some difficulty.

The patient having been etherized on former occasions for slight surgical operations, by the reporter, without any ill consequences, and being at this time in perfect health, M. de Confevron yielded to her solicitations, not regarding some slight mental emotion which she had shortly before experienced, as presenting a sufficient obstacle to the gratification of her wishes.

Having determined to produce only the slightest degree of insensibility, about fifteen grains (one gramme) of chloroform was poured upon a fold of lint the size of a filbert, enclosed in a handkerckief. This was held at a distance from the nostrils by the patient herself.

Its effects were manifest in eight seconds, and the reporter remarked constant winking of the eyelids. The patient repulsed the dentist's hands, making signs that the effect was not complete. She then made four or five fuller inspirations. At that instant M. de Confevron removed the handkerchief, and only took his eyes from the patient for one moment, occupied by placing them on the nearest piece of furniture; but in this short interval he found the patient's face turned pale, the lips discolored, the features altered, the eyes turned upward, the pupils widely dilated, the jaw closed, the head drawn backwards, the pulse not to be felt, the limbs all relaxed; and a few inspirations at long intervals were the only remaining indications of life.

For two hours every means of restoration were employed:—stimulation of the nostrils by ammonia; frictions of the surface; actual cauterization of the præcordial region; artificial respiration and galvanism—all were had recourse to, but without success. The patient was dead.

An examination was made of the body thirty-eight hours after death.—The membranes of the brain, and especially the veins of the base of the cranium, were gorged with black fluid blood. The sinuses of the *dura mater* were also full of blood. The substance of the brain was healthy, and when cut presented very numerous dark bloody points. A considerable quantity of serum filled the base of the cranium and vertebral canal. A perceptible quantity of air bubbles was seen in all the veins at the base of the brain. The heart was flaccid, and a considerable portion of black fluid blood mixed with air-bells escaped from a puncture made into the left auricle. Air-bells were also found in all the large veins of the body. The lungs were crepitant throughout, and presented, when cut, a greyish slate color. The abdomen was distended by gases. The intestines were not examined.

*Remarks.*—"This case, possessing of itself a high degree of interest, might be regarded as an isolated fact — as one of those unfortunate cases occasionally occurring, but from which science derives but little instruction. It might be said to be one of those rare and inexplicable instances of sudden death from unknown causes, of which other examples may be found in both ancient and modern writers. But viewed, on the other hand, in connection with the cases related by Mess. Robert, Barrier and Gorre, it acquires great importance and leads to very different conclusions. It may be observed, in the first place, that facts of this kind have become so multiplied that it is no longer possible to attribute them to any other cause than the operation of the chloroform. Omitting all the cases of which the exact details are not given, and confining the attention to those already referred to, it is clearly impossible to arrive at any other conclusion. In all these, the symptoms which precede death, compared with the necroscopic results, prove the extinction of life to have been owing to real asphyxia, the direct effect of the special deleterious influence of chloroform on the brain."

"In the present instance the patient died as if struck by lightning, despite the small quantity of the vapor inhaled, and the precautions observed. There was no warning, as in M. Gorre's case, no complaint of sense of suffocation; on the contrary, the patient, at the moment of expiring, indicated that the anæsthesia was not complete, and this was shown by her still tightly holding the handkerchief, when taken from her.

"M. Sedillot has shown, that the supervention of muscular relaxation is the period at which the administration of the agent should cease; but the preceding case shows that this indication, since, in M. Barrier's case, life and the pulse ceased simultaneously.

"The presence of air in the vessels, M. de Confevron is disposed to attribute rather to his own forcible efforts to produce artificial respiration, than to the spontaneous development of air in the veins as a peculiar effect of *ether*, as supposed by M. Gorre. The other pathological appearances were distinctly those of asphyxia, resulting from a poisonous influence exerted on the brain."

II. *Instance of Death from the Use of Chloroform administered during a Surgical Operation.* By Mr. Samuel Solly, Surgeon to St. Thomas's Hospital. London Medical Gazette, No. 1144, Vol. XLIV. 2d Nov. 1849, page 757.

John Shorter, aged 48, a porter, known to Mr. Solly for some time as a very active messenger, habits intemperate, but apparently in perfect health, was admitted into George's ward, under Mr. Solly, on the 9th of October, 1849, suffering from onychia of the left great toe, which had existed for some time. It was determined to remove the nail, the man having decided before entering the Hospital on taking chloroform

On Wednesday, Oct. 10, at a quarter to 2, P. M., he began to inhale the chloroform with one dram in the inhaler. It had no visible effect for about two minutes. It then excited him, and the instrument was removed from his mouth, and about ten drops more were added. He then became almost immediately insensible; the chloroform was taken away, and the nail removed. He continued insensible; and, his face becoming dark, the pulse small, quick but regular, respiration laborious, his neckerchief was removed, and his chest exposod to the fresh air from a window close to the bed; cold water was dashed in his face, the chest rubbed, and ammonia applind to the nose. After struggling for about a minute, he became still, the skin cold, pulse scarcely perceptible, and soon ceased to be found at the wrist; respiration became slow and at intervals, but continued a few seconds after the cessation of the pulse. Immediately on the appearance of these symptoms, artificial respiration was commenced by depressing the ribs with the hands, and then allowing them to rise again until the proper apparatus was brought, when respiration was kept up by means of the trachea-tube and bellows, and oxygen gas introduced into the lungs by the same means. Galvanism was also applied through the heart and diaphragm, but all signs of life ceased about six or seven minutes after the commencement of inhalation. These means were persisted in until a quarter past 3, but to no purpose. On removing the inhaler, the sponge, which contained only one drachm, fell upon the floor, and the chloroform splashed about — thus showing that a considerable part of the chloroform remained unused; so that the patient could not have inhaled more than a drachm. Every effort was made to procure a *post-mortem* examination, but in vain.

III. *Instance of fatal termination of Delirium Tremens during the administration of Chloroform.* London Medical Gazette, Vol. XLIV., No. 23, 1849.

The particulars of this case may be found in No. 26 of the last Vol. of this Journal.

IV. *Case of Death ascribed to Inhalation of Chloroform.*

In the Hereford Journal is recorded a death from the use of chloroform, previous to the performance of a surgical operation. On Wednesday, Dec. 5, a young Welsh woman, named Jones, living near the Craven Arms, between Church Stretton and Ludlow, had come to Shrewsburry to undergo the operation of extirpation of the eye-ball. Mr. W. J. Clement, the surgeon under whose care she was, administered previously about one third of the quantity of chloroform, It is said, which he was in the habit of giving to other patients. The patient very soon became insensible, and, after muttering a few words in Welsh, expired almost immediately, as if she had been poisoned by hydrocyanic acid.

On the evening of the same day, the Coroner held an inquest on the body; and the jury returned a verdict of "Died by apoplexy, in consequence of inhaling one drachm of chloroform." J. C. W.

*Boston, Feb.* 9, 1850.

